# Supplementary material for: Untargeted Metabolome Analysis Reveals Reductions in Maternal Hepatic Glucose and Amino Acid Content That Correlate with Fetal Organ Weights in a Mouse Model of Fetal Alcohol Spectrum Disorders
Source: Nutrients. 2022 Mar 5;14(5):1096. doi: 10.3390/nu14051096 (PMC8912878; doi:10.3390/nu14051096)
Supplement: Supplementary file 1 [file nutrients-14-01096-s001.zip › nutrients-1613448-Supplementary-1.pdf]

Untargeted Metabolome Analysis Reveals Reductions in Maternal Hepatic Glucose and Amino Acid Content That Correlate with Fetal Organ Weights in a Mouse Model of Fetal Alcohol Spectrum Disorders

Nipun Saini [1,*], Manjot S. Virdee [1], Kaylee K. Helfrich [1,2], Sze Ting Cecilia Kwan [1], Sandra M. Mooney [1,2] and Susan M. Smith [1,2,*]

Supplementary Methods.

1.1. Metabolite Analysis

A global metabolomics approach was performed using a proprietary pipeline developed by Metabolon Inc. (Durham, NC). Samples were transported to Metabolon on dry ice and immediately stored at -80°C until processing. Samples were rapidly thawed, methanol containing several recovery standards was added proportionate to tissue weight, and the samples dissociated under vigorous shaking (2min, GenoGrinder 2000, Glen Mills) to denature the protein and release protein-bound small molecules. Protein concentrations were determined using the Bradford method for later data normalization. Samples were centrifuged to remove the protein precipitate, and the methanol extracts were harvested using an automated MicroLab STAR system (Hamilton Company, Salt Lake City, UT). Each sample was divided into five aliquots for their subsequent respective analyses, placed briefly on a TurboVap (Zymark) to remove the organic solvent, then stored overnight under nitrogen. Samples were reconstituted in the appropriate solvent, optimized for that analytical mode, and containing standards at fixed concentrations to normalize injection volume and chromatographic consistency.

Liquid chromatography-mass spectrometry (LC-MS) analysis was carried out using a Waters ACQUITY ultra high-performance liquid chromatography (UHPLC) and a Thermo Scientific Q-Exactive orbitrap mass spectrometer interfaced with a heated electrospray ionization source and Orbitrap mass analyzer operated at 35,000 mass resolution, as detailed in [1]. Two samples were subjected to reverse phase (RP)/UPLC-MS/MS methods with positive ion mode electrospray ionization (ESI). In the first, which targeted more hydrophilic compounds, the extract was gradient eluted from a C18 column (Waters UPLC BEH C18-2.1 × 100mm, 1.7um) using water and methanol, and containing 0.05% perfluoropentanoic acid (PFPA) and 0.1% formic acid. The second positive ion mode ESI targeted more hydrophobic compounds, using the same C18 column with 0.05% PFPA and 0.1% formic acid in water, but gradient eluted using 50% methanol and 50% acetonitrile. A third aliquot was analyzed using RP/UPLC-MS/MS with negative ion mode ESI, using the same C18 column with 6.5mM ammonium bicarbonate at pH 8 in water, and gradient eluted using 95% methanol/5% water with 6.5mM ammonium bicarbonate at pH 8. The fourth sample was analyzed using HILIC/UPLC-MS/MS with negative ion mode ESI, using a Waters HILIC column (Waters UPLC BEH amide 2.1 × 150mm, 1.7um) with 15% water/5% methanol/80% acetonitrile against a gradient of 50/50 water and acetonitrile, all containing 10mM ammonium formate, pH 10.8. The fifth sample was reserved for backup. The separation and run times were ~7min for the NEG and HILIC, and ~3.5min for the POSEarly and POSLate, and used an alternating column system wherein one column performed the separation while the other was being cleaned and reconditioned for the next sample. The MS analysis alternated between MS and data-dependent MS scans using dynamic exclusion. The scan range varied slightly between methods but covered 70 – 1000 m/z.

Quality controls (QC) included a process blank of ultrapure water, a solvent blank, and a pooled matrix sample comprised of a small aliquot from each experimental sample. Five QC samples and three

process blank samples were processed for every batch of 30 samples. Added to each experimental sample was a recovery sample cocktail of isotopically labeled and halogenated compounds selected to not interfere with measurement of endogenous samples, and used to aid chromatographic alignment and monitor instrument performance, as detailed [1]. As an additional quality control, the overall process variability was determined by calculating the median relative standard deviation (RSD) value for all endogenous metabolites present in 100% of the QC samples, which were technical replicates created from the pool of experimental samples. The median RSD for these QC samples was 8%. The injection order of the experimental samples was randomized, and the quality control samples were evenly interspersed between these injections. The samples analyzed here were all run in a single day. Additional details on the analytical methodology are presented in [1], which reports an average intra-assay coefficient of variation (CV) of < 7%, and an average inter-assay CV of 9.9% – 12.6%.

1.2. Data Analysis

Raw data were extracted, peak-identified, and the quality controls processed using custom software proprietary to Metabolon and based on criteria of peak detection, integration, and alignment [2,3]. Biochemicals were identified by comparison to Metabolon's proprietary library, built from the analysis of authentic standards and comprised of 3300 purified, authenticated compounds annotated with respect to retention time/index (RI), mass-to-charge ratio (*m/z*), and chromatographic data including MS/MS spectral data on all four platforms [2], consistent with Tier 1 identification standards defined by the Metabolomics Standards Initiative [4]. Feature alignment across the samples was accomplished using a series of internal standards to establish a retention index (RI) ladder based upon the internal instrument performance standards. RIs of experimental peaks were determined by (i) comparison against the internal standard RI markers within 150 RI units (~10 seconds) and assumed a linear fit, (ii) mass match to the library authenticated standard within +/- 10ppm, and (iii) quality of the fragmentation spectrum match between the experimental and library compound [1]. The use of all three criteria were used to distinguish and differentiate the biochemicals. All compounds reported were manually reviewed by a QC analyst to confirm the quality of peak integration, alignment, and identification across all samples in the study. Signals representing system artifacts, mis-assignments, and background noise were removed. Peaks were quantified using area-under-the-curve, and values are presented as median scaled data.

References:

1. Ford, L.; Kennedy, A.D.; Goodman, K.D.; Pappan, K.L.; Evans, A.M.; Miller, L.A.D.; Wulff, J.E.; Wiggs, B.R.; Lennon, J.J.; Elsea, S.; et al. Precision of a Clinical Metabolomics Profiling Platform for Use in the Identification of Inborn Errors of Metabolism. *J Appl Lab Med* **2020**, *5*, 342-356, doi:10.1093/jalm/jfz026.
2. Dehaven, C.D.; Evans, A.M.; Dai, H.; Lawton, K.A. Organization of GC/MS and LC/MS metabolomics data into chemical libraries. *J Cheminform* **2010**, *2*, 9, doi:10.1186/1758-2946-2-9.
3. DeHaven, C.D.E., A.M; Dai, H and Lawton, K.A. Software Techniques for Enabling High-Throughput Analysis of Metabolomic Datasets. In *Metabolomics*, Roessner, U., Ed.; InTech Open; 2012.
4. Sumner, L.W.; Amberg, A.; Barrett, D.; Beale, M.H.; Beger, R.; Daykin, C.A.; Fan, T.W.; Fiehn, O.; Goodacre, R.; Griffin, J.L.; et al. Proposed minimum reporting standards for chemical analysis Chemical Analysis Working Group (CAWG) Metabolomics Standards Initiative (MSI). *Metabolomics* **2007**, *3*, 211-221, doi:10.1007/s11306-007-0082-2.

Supplementary Figures (Figure S1–S6), Supplementary Tables (Table S1–S3)

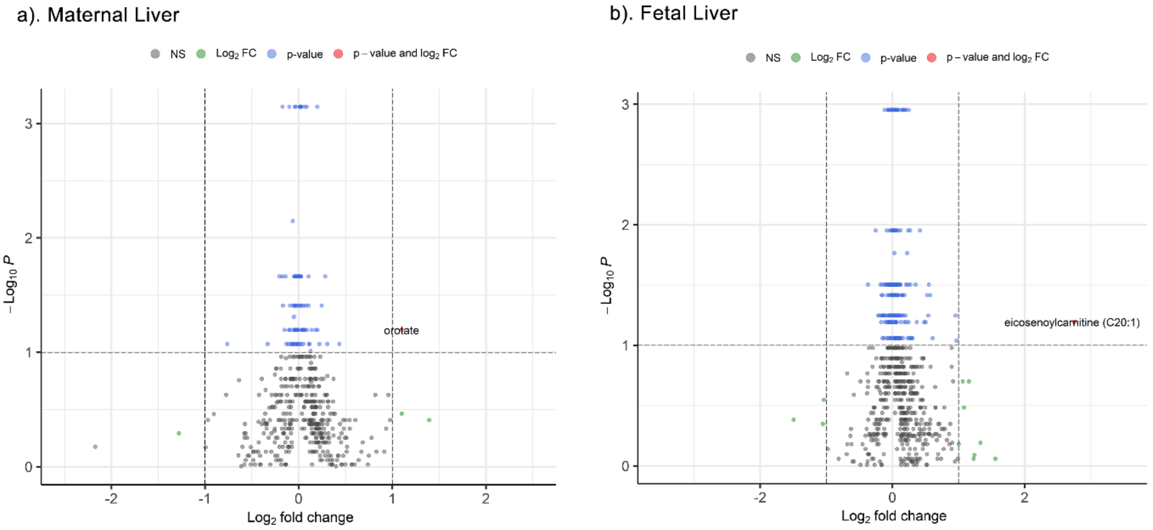

Figure S1. The Volcano plot for the analysis of 724 metabolites in (**a**) Maternal liver and (**b**) Fetal liver at q < 0.05.

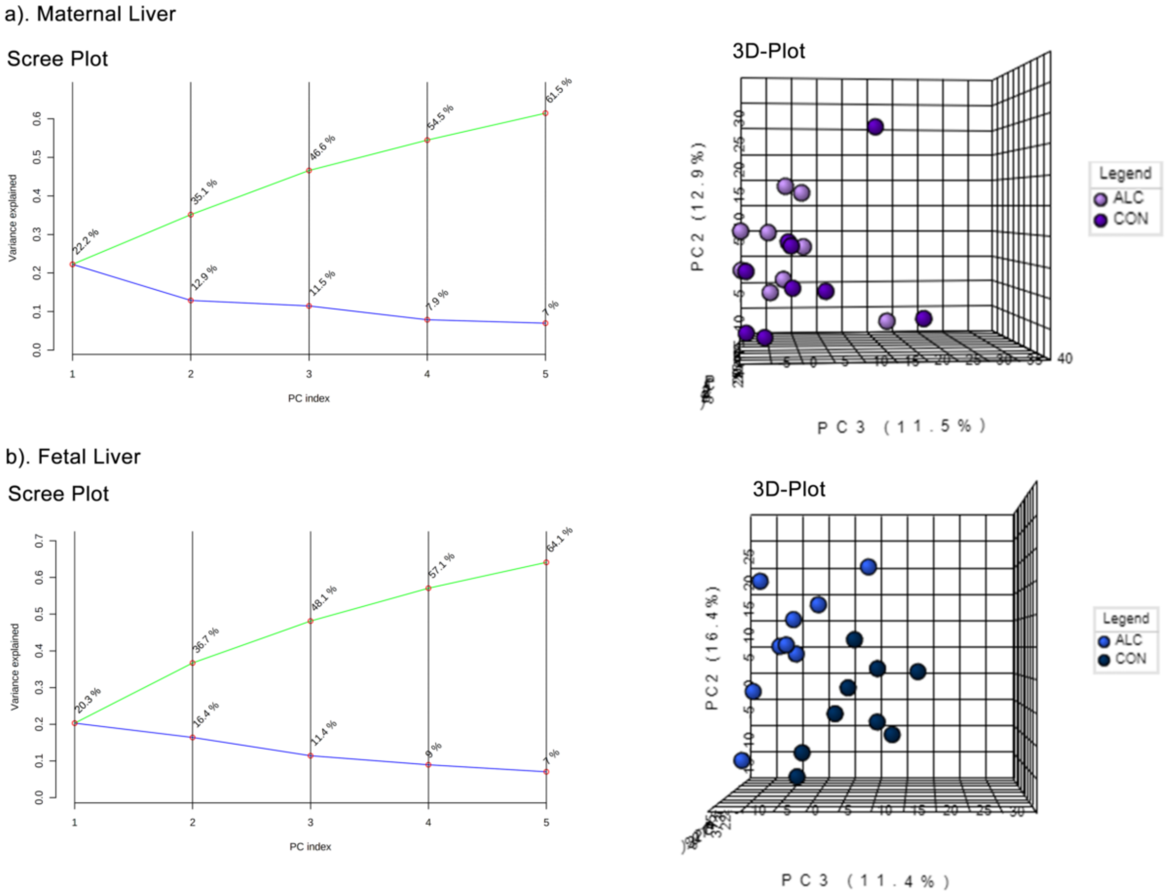

Figure S2. Scree plot and 3D-plot of the PCA analysis in (**a**) Maternal Liver and (**b**) Fetal liver. *N* = 724 metabolites.

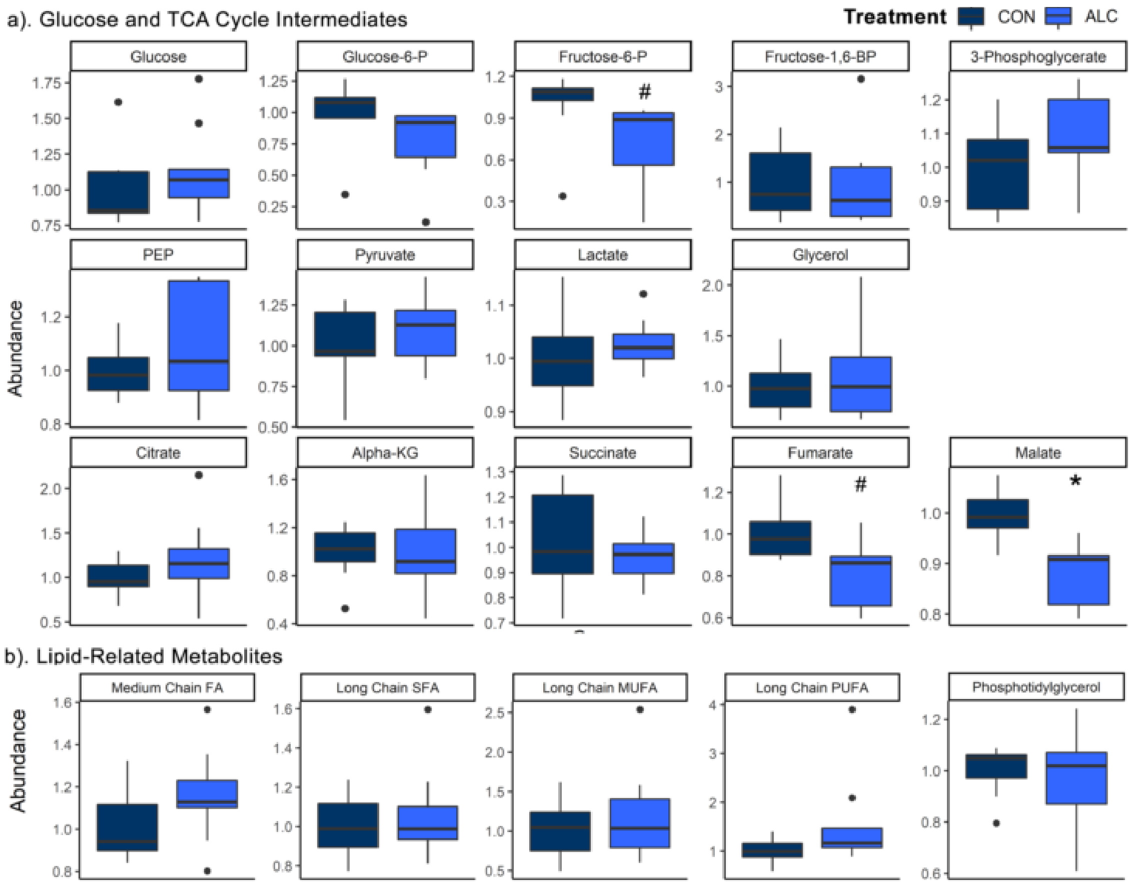

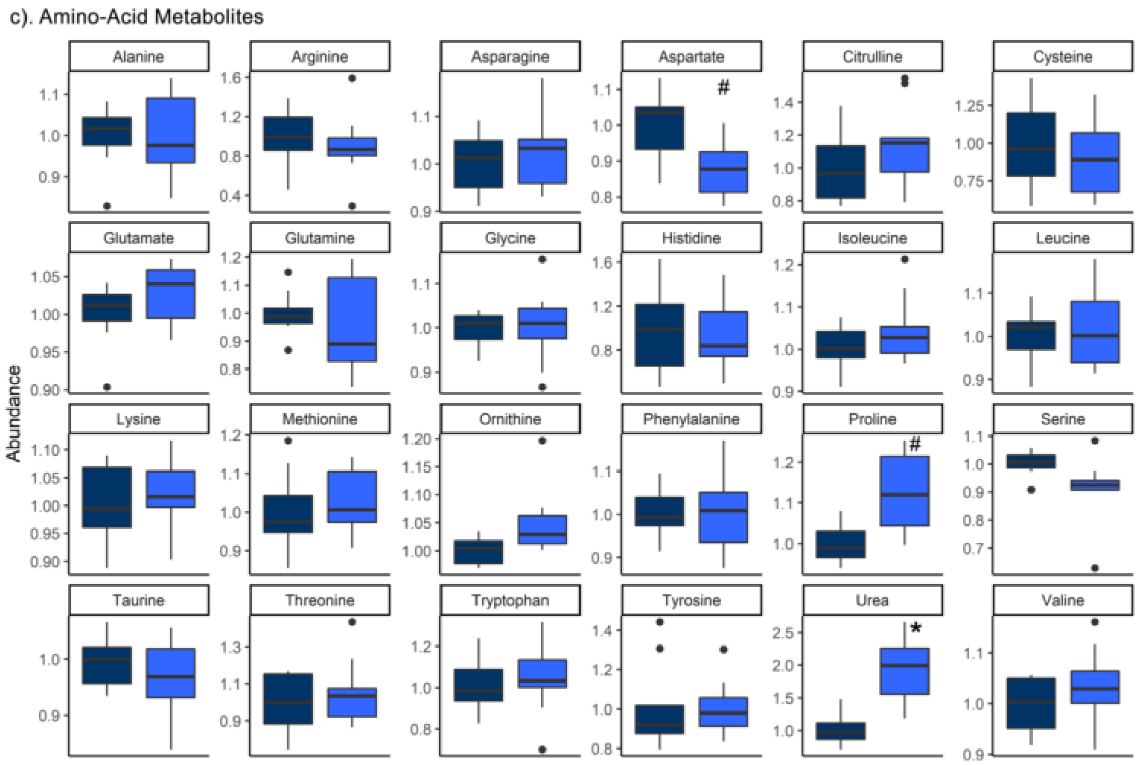

Figure S3. Relative Abundance of (**a**) Glucose and TCA Cycle Intermediates, (**b**) Lipid Classes, and (**c**) Amino-Acid Related Metabolites in ALC vs. CON in Fetal Liver. Lipid classes are: medium chain FAs (C6:0-C12:0), saturated FAs (C14:0-C22:0), monounsaturated FAs (C14:1-C22:1), polyunsaturated FAs (C14:2-C24:6), and phosphotidylglycerol (C16:0, C18:0, C18:1 and C18:2 at sn1 or sn2 position). Abundance of CON is normalized to 1.0, and comparisons used Wilcoxon test. Boxplots depict the data's spread (measured in inter quartile range), middle line depicts the median, and the dots indicate outliers. * $q \leq 0.05$, # $0.05 > q \leq 0.10$. Alpha-KG alpha-ketoglutarate; FA Fatty Acid; Fructose-6-P fructose-6-phosphate; Fructose-1,6-BP fructose-1,6-bisphosphate; Glucose-6-P glucose-6-phosphate; MUFA monounsaturated FA; PEP phosphoenolpyruvate; PUFA polyunsaturated FA; SFA saturated FA.

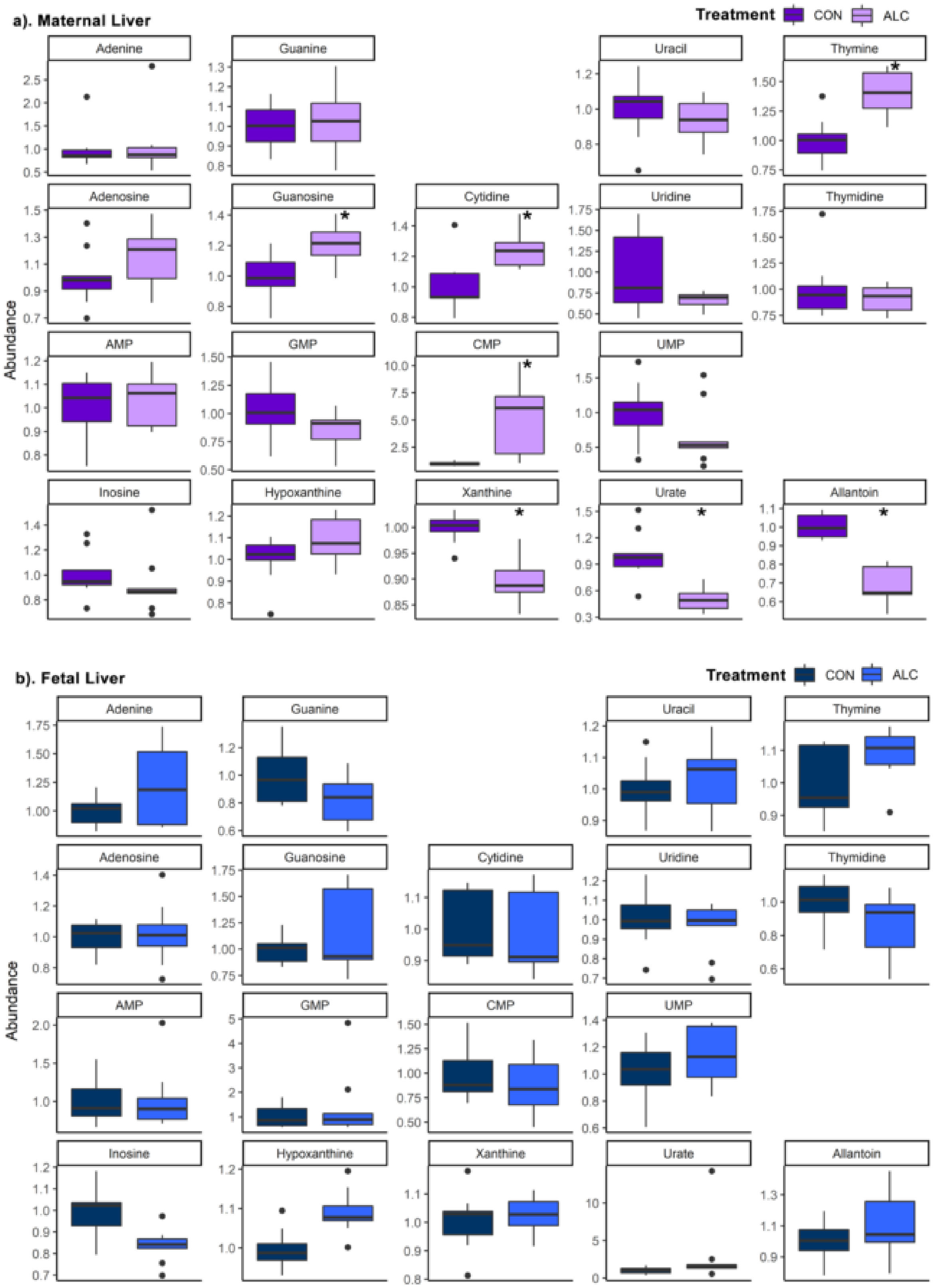

a). Maternal Liver
Treatment CON ALC
Adenine
Guanine
Uracil
Thymine
Adenosine
Guanosine
Cytidine
Uridine
Thymidine
AMP
GMP
CMP
UMP
Inosine
Hypoxanthine
Xanthine
Urate
Allantoin
Abundance
b). Fetal Liver
Treatment CON ALC
Adenine
Guanine
Uracil
Thymine
Adenosine
Guanosine
Cytidine
Uridine
Thymidine
AMP
GMP
CMP
UMP
Inosine
Hypoxanthine
Xanthine
Urate
Allantoin
Abundance

Figure S4. Relative Abundance of Purine and Pyrimidine Metabolites in ALC vs. CON in (**a**) Maternal and (**b**) Fetal Liver. Abundance of CON is normalized to 1.0, and comparisons used Wilcoxon test. Boxplots depict the data's spread (measured in inter quartile range), middle line depicts the median, and the dots indicate outliers. * q ≤ 0.05. AMP adenosine-monophosphate; GMP guanine-monophosphate; CMP cytosine-monophosphate; UMP uridine-monophosphate. Note that TMP was not detected.

a). Plots separating Maternal Liver Metabolites based on Fetal Body Weights

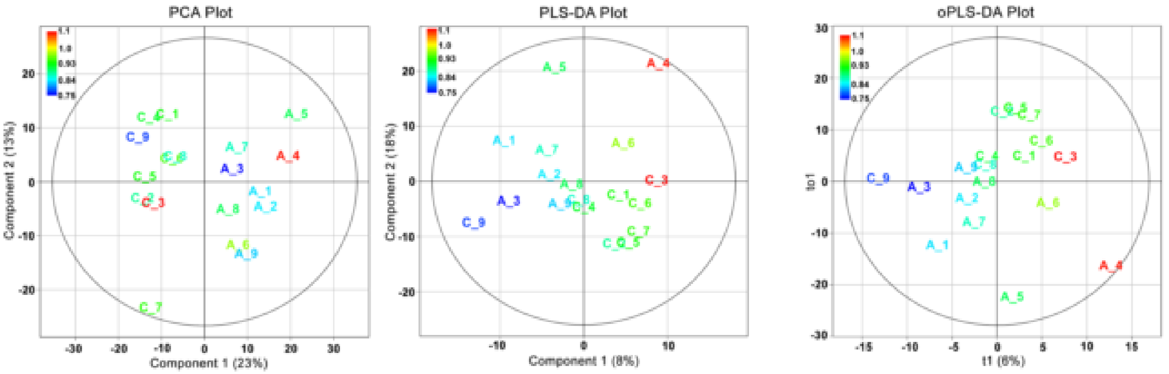

b). Top 30 metabolites identified in oPLSDA

Top 30 metabolites	VIP Scores	FC (ALC/CON)	*q-value*	*Pearson's Correlation*	
				R-value	*p-value*
3'-AMP	2.811	0.98	0.9500	-0.55	***0.0180***
Stearoyl-linoleoyl-glycerol (18:0/18:2) [2]	2.779	1.02	1.0000	0.62	***0.0063***
Beta-muricholate	2.633	0.88	0.7800	-0.52	***0.0260***
1-stearoyl-2-linoleoyl-GPE (18:0/18:2)	2.572	0.82	0.5000	0.62	***0.0057***
Tauro-beta-muricholate	2.496	1.18	0.6700	-0.45	*0.0580*
N-stearoyl-sphingosine (d18:1/18:0)	2.491	1.10	0.3800	0.51	***0.0310***
Creatine	2.456	1.24	0.1000	-0.61	***0.0067***
Dihomo-linoleoylcarnitine (C20:2)	2.400	0.75	0.5800	0.39	0.1112
Gamma-glutamyl-epsilon-lysine	2.395	1.01	0.7300	-0.42	*0.0792*
N6,N6-dimethyllysine	2.389	0.92	0.2800	0.53	***0.0232***
N-formylmethionine	2.358	1.05	0.5400	-0.47	***0.0490***
X - 16060	2.340	0.99	0.9500	-0.46	*0.0562*
Stearoyl sphingomyelin (d18:1/18:0)	2.273	1.05	0.6300	0.50	***0.0350***
Malonate	2.260	0.89	*0.0800*	0.66	***0.0031***
Glycosyl-N-stearoyl-sphingosine (d18:1/18:0)	2.190	0.95	0.8200	0.47	*0.0519*
1-palmitoyl-2-linoleoyl-GPC (16:0/18:2)	2.183	0.93	0.3400	0.47	*0.0504*
N-palmitoylglycine	2.171	1.13	0.7300	0.28	0.2588
X - 10457	2.164	1.09	0.4100	0.53	***0.0241***
Glycosyl-N-arachidoyl-sphingosine (d18:1/20:0)	2.146	0.84	0.1600	0.36	0.1394
1-stearoyl-2-arachidonoyl-GPS (18:0/20:4)	2.141	0.96	0.6700	0.46	*0.0540*
X - 16580	2.132	0.98	1.0000	0.51	***0.0292***
Cytidine 2' or 3'-monophosphate (2' or 3'-CMP)	2.126	1.94	0.5800	-0.42	*0.0803*
N-stearoyl-sphinganine (d18:0/18:0)	2.121	1.14	0.6700	0.04	*0.0985*
Oleoyl-arachidonoyl-glycerol (18:1/20:4) [1]	2.099	1.20	0.4100	0.32	0.1893
1-methylnicotinamide	2.075	0.72	*0.0800*	-0.44	*0.0704*
2'-deoxyguanosine	2.055	1.10	0.1800	-0.44	*0.0702*
Linoleoyl ethanolamide	2.049	1.04	0.8600	0.47	***0.0495***
Ceramide (d18:1/17:0, d17:1/18:0)	2.043	1.03	0.8600	0.43	*0.0780*
1-linoleoyl-GPE (18:2)	2.035	1.26	0.0300	0.40	*0.0958*
Hydroxypalmitoyl sphingomyelin (d18:1/16:0(OH))	2.031	1.11	0.4500	-0.41	*0.0953*

c). Fetal Body Weight Correlations

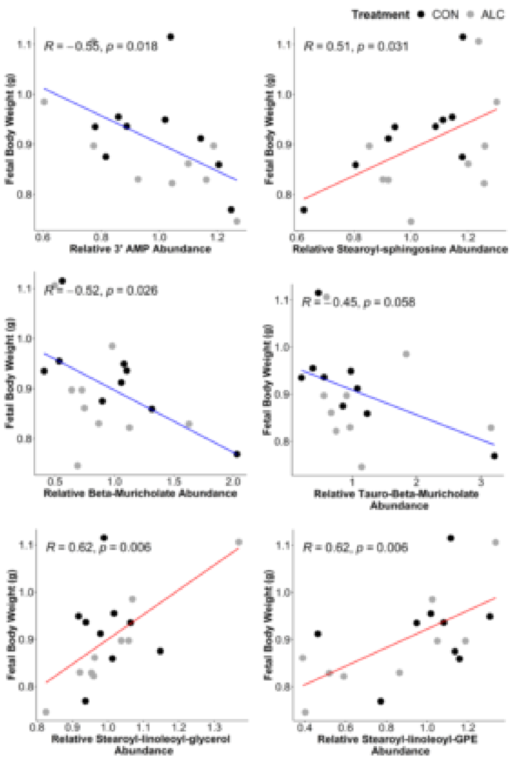

Figure S5. Separation of Maternal Liver metabolites as ALC vs. CON Based on Fetal Body Weights. (**a**) PCA, PLS-DA and oPLSDA plots depicting the separation of maternal liver metabolites as ALC vs. CON based on Fetal Body Weights. The legend and the corresponding color in the plots indicate increase in fetal body weight (in g) values from blue to red. (**b**) The Top 30 Metabolites extracted as VIP scores in the first orthogonal component in oPLSDA with FC (ALC/CON), q-value, and Pearson's Correlation (R)

analysis with respect to Fetal Body Weights. Significant correlations at $p \leq 0.05$ are bold and italicized, and $0.05 < p \leq 0.10$ are italicized. (**c**) Correlation Plots of top select metabolites identified in oPLSDA with Fetal Body Weights. The blue and red trendlines indicate significant negative and positive correlations, respectively.

a). Plots separating Fetal Liver Metabolites based on Fetal Body Weights

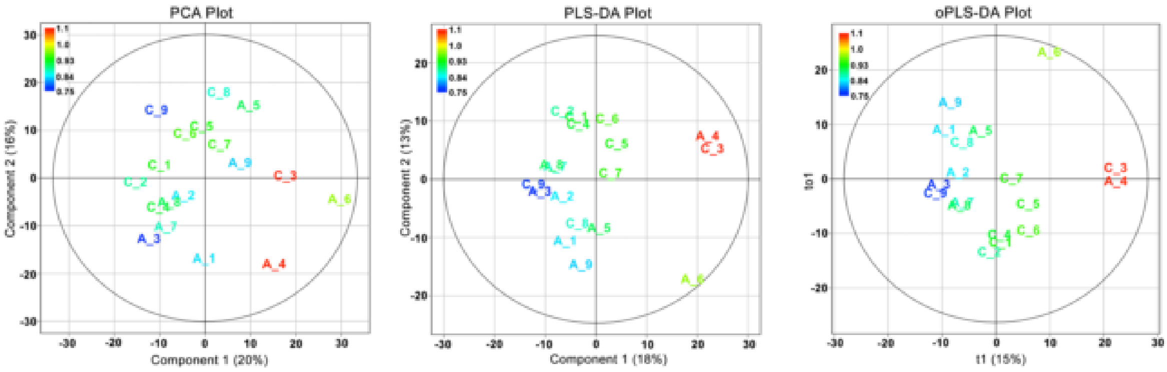

b). Top 30 metabolites identified in oPLSDA

Top 30 Metabolites	VIP Scores	FC (ALC/CON)	*q-value*	*Pearson's Correlation*	
				R-value	*p-value*
5-hydroxyindoleacetate	2.314	0.88	0.3858	0.85	***0.0000***
Formiminoglutamate	2.305	0.75	0.2744	0.89	***0.0000***
N2,N2-dimethylguanosine	2.261	1.05	0.6748	-0.79	***0.0001***
N-acetylglucosamine 6-phosphate	2.237	0.95	0.9304	-0.79	***0.0001***
2-hydroxyglutarate	2.151	0.85	0.2451	0.79	***0.0001***
X - 24665	2.127	1.12	0.7069	0.75	***0.0003***
Biliverdin	2.126	0.77	0.6005	0.80	***0.0001***
N-acetyl-glucosamine 1-phosphate	2.118	0.95	0.9155	-0.77	***0.0002***
2'-O-methyluridine	2.115	1.00	0.8777	-0.73	***0.0007***
Maltotriose	2.101	0.78	0.1587	0.76	***0.0003***
X - 15136	2.097	1.02	0.9155	0.78	***0.0002***
N-acetylserine	2.062	1.13	0.6320	-0.61	***0.0069***
1-ribosyl-imidazoleacetate	2.060	0.72	0.1048	0.76	***0.0003***
Ribose 5-phosphate	2.046	0.82	0.4329	-0.71	***0.0010***
Phenylacetylglycine	2.045	0.84	0.3858	0.76	***0.0003***
X - 23710	2.039	1.09	0.9304	0.79	***0.0001***
Gamma-glutamylleucine	2.019	1.02	0.9155	-0.68	***0.0018***
Glycosyl-N-stearoyl-sphingosine (d18:1/18:0)	2.015	0.88	0.5207	0.75	***0.0003***
1-myristoyl-2-palmitoyl-GPC (14:0/16:0)	2.013	1.09	0.2732	-0.73	***0.0006***
X - 25055	2.006	1.19	0.6320	-0.61	***0.0070***
Pyridoxamine	2.002	1.44	0.1048	-0.65	***0.0038***
Gamma-glutamylphenylalanine	1.979	0.89	0.9155	-0.71	***0.0009***
Mannose 6-phosphate	1.955	0.79	0.2862	-0.72	***0.0008***
X - 24697	1.954	1.11	0.7853	-0.64	***0.0043***
Quinolinate	1.950	0.93	0.8777	-0.60	***0.0079***
Mannose	1.948	1.24	0.5622	0.75	***0.0003***
Glucose 6-phosphate	1.938	0.77	0.1587	-0.69	***0.0015***
Methionine sulfoxide	1.937	1.36	0.3858	-0.62	***0.0056***
Riboflavin (Vitamin B2)	1.931	0.93	0.9304	-0.72	***0.0008***
Taurocholate	1.928	1.04	0.6320	0.72	***0.0008***

c). Fetal Body Weight Correlations

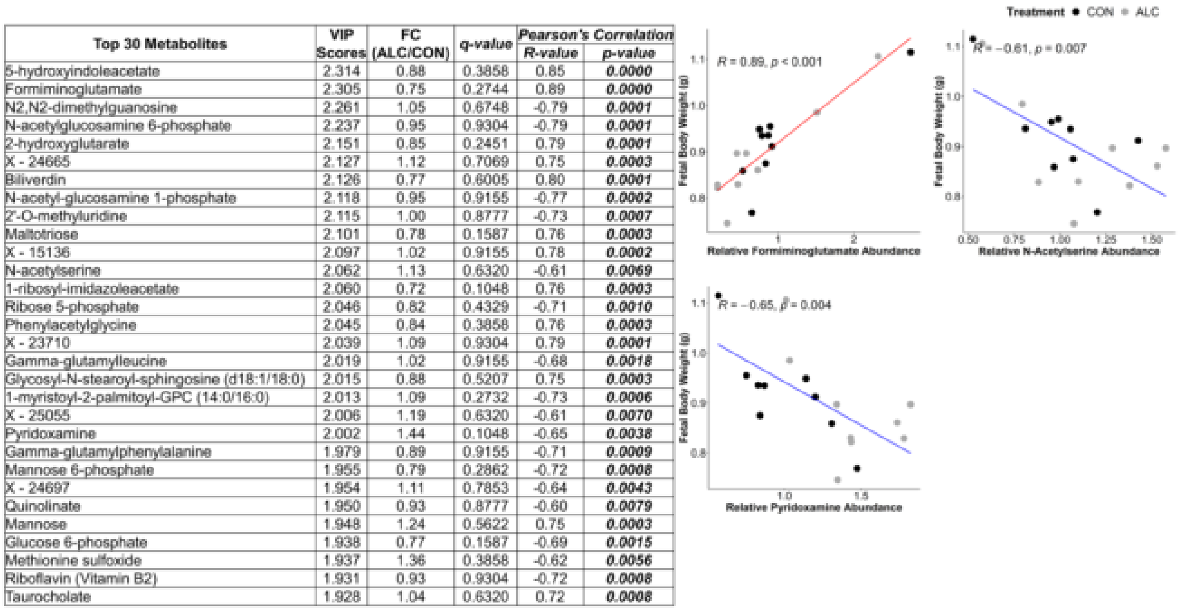

Figure S6. Separation of Fetal Liver Metabolites as ALC vs. CON Based on Fetal Body Weights. (**a**) PCA, PLS-DA and oPLSDA plots depicting the separation of Fetal liver metabolites as ALC vs. CON based on Fetal Body Weights. The legend and the corresponding color in the plots indicate increase in fetal body weight (in g) values from blue to red. (**b**) The Top 30 Metabolites extracted as VIP scores in the first orthogonal component in oPLSDA with FC (ALC/CON), q-value, and Pearson's Correlation (R) analysis with respect to Fetal Body Weights. Significant correlations at $p \leq 0.05$ are bold and italicized. (**c**) Correlation Plots of Top Select Metabolites Identified in oPLSDA with Fetal Body Weights. The blue and red trendlines indicate significant negative and positive correlations, respectively.

Table S1. Maternal and Fetal Weight and Survival Characteristics.

	**Controls**	**Alcohol-Exposed**	***p*-Value**
Maternal Weight at E0.5, g	18.37 ± 1.18	18.16 ± 1.09	0.71
Maternal Weight at E8.5, g	21.20 ± 1.68	21.10 ± 1.36	0.88
Maternal Weight Gain (E0.5-E17.5), g	13.96 ± 2.23	13.28 ± 2.25	0.52
Maternal Weight Gain (E8.5-E17.5), g	11.08 ± 1.50	10.35 ± 1.47	0.28
Litter Size at E17.5	7.00 ± 1.22	7.11 ± 1.36	0.85
Resorptions at E17.5	1.00 ± 1.00	1.00 ± 1.22	0.81
Percent Survival at E17.5 *	88.8 ± 3.58	84.7 ± 4.57	0.69
Fetal Weight at E17.5, g	0.92 ± 0.11	0.88 ± 0.12	0.31
Fetal Liver Weight at E17.5, g	0.049 ± 0.004	0.040 ± 0.004	<0.001
Fetal Brain Weight at E17.5, g	0.052 ± 0.004	0.044 ± 0.003	<0.001
Fetal Liver/ Body Weight Ratio	0.053 ± 0.004	0.045 ± 0.005	<0.01
Fetal Brain/ Body Weight Ratio	0.056 ± 0.004	0.050 ± 0.007	0.07

All values are mean ± SD. with N=9 dams per treatment group. Statistical comparisons using ANOVA (for normal data with equal variance) or Kruskal-Wallis test (for non-normal data and/or data with unequal variance).*Percent Survival calculated as [Number of live fetuses / (number of live fetuses + number of resorptions)] × 100.

Table S2. List of Metabolites Significantly Altered by Alcohol Exposure in Maternal Liver with their Identifiers, Relative Abundance Fold-change (ALC/CON) and *q-values.*

Table S3. List of Metabolites Significantly Altered by Alcohol Exposure in Fetal Liver with their Identifiers, Relative Abundance Fold-change (ALC/CON) and *q-values*.
